# Using Networks To Understand Medical Data: The Case of Class III Malocclusions

**DOI:** 10.1371/journal.pone.0044521

**Published:** 2012-09-21

**Authors:** Antonio Scala, Pietro Auconi, Marco Scazzocchio, Guido Caldarelli, James A. McNamara, Lorenzo Franchi

**Affiliations:** 1 Istituto dei Sistemi Complessi Udr “La Sapienza”, Roma, Italy; 2 London Institute of Mathematical Sciences, London, United Kingdom; 3 Private Practice of Orthodontics, Rome, Italy; 4 NATWORKS Unit for the Study of Natural Networks, IMT Lucca Institute for Advanced Studies, Lucca, Italy; 5 Department of Orthodontics and Pediatric Dentistry, School of Dentistry, The University of Michigan, Ann Arbor, Michigan, United States of America; 6 Department of Cell and Developmental Biology, School of Medicine, The University of Michigan, Ann Arbor, Michigan, United States of America; 7 Center for Human Growth and Development, The University of Michigan, Ann Arbor, Michigan, United States of America; 8 Private Practice of Orthodontics, Ann Arbor, Michigan, United States of America; 9 Department of Orthodontics, The University of Florence, Florence, Italy; Universidad Carlos III de Madrid, Spain

## Abstract

A system of elements that interact or regulate each other can be represented by a mathematical object called a network. While network analysis has been successfully applied to high-throughput biological systems, less has been done regarding their application in more applied fields of medicine; here we show an application based on standard medical diagnostic data. We apply network analysis to Class III malocclusion, one of the most difficult to understand and treat orofacial anomaly. We hypothesize that different interactions of the skeletal components can contribute to pathological disequilibrium; in order to test this hypothesis, we apply network analysis to 532 Class III young female patients. The topology of the Class III malocclusion obtained by network analysis shows a strong co-occurrence of abnormal skeletal features. The pattern of these occurrences influences the vertical and horizontal balance of disharmony in skeletal form and position. Patients with more unbalanced orthodontic phenotypes show preponderance of the pathological skeletal nodes and minor relevance of adaptive dentoalveolar equilibrating nodes. Furthermore, by applying Power Graphs analysis we identify some functional modules among orthodontic nodes. These modules correspond to groups of tightly inter-related features and presumably constitute the key regulators of plasticity and the sites of unbalance of the growing dentofacial Class III system. The data of the present study show that, in their most basic abstraction level, the orofacial characteristics can be represented as graphs using nodes to represent orthodontic characteristics, and edges to represent their various types of interactions. The applications of this mathematical model could improve the interpretation of the quantitative, patient-specific information, and help to better targeting therapy. Last but not least, the methodology we have applied in analyzing orthodontic features can be applied easily to other fields of the medical science.

## Introduction

A general way to understand complex biological systems is to represent them using the simplest units of architecture. Such patterns of local and global interconnection are called *networks*. A network, or in more formal mathematical language, a *graph*, is a simplified representation that reduces a system to an abstract structure capturing the basis of connection pattern of the system [1], [2]. The simplest possible network representation reduces the system's elements to *nodes* (“*vertices*”) and their pairwise relationships to *links* (“*edges*”) connecting pairs of nodes. Links represent functional interactions or anatomical relationships between the nodes, such as “catalyze”, or “binds to”, or “is converted to”, or “shift” [3], [4]. The network's inference and analysis refers to information on the identity and the state of the elements of a system to their functional relationships and to the extraction of biological insight and predictions. A multitude of studies have shown that meaningful biological properties can be extracted by network analysis [5], [6].

An important advancement in network science has been the possibility of identifying and localizing sub-networks of functional modules (*motifs*) in complex systems [7]. The decomposition of large networks into distinct components, or modules, has to be regarded as a major approach to deal with the complexity of large biological networks. A motif refers to a group of physically or functionally connected components (nodes in graph) that work together to achieve the desired biological function. These organized sets of interactions are capable of local ordering, function, process information, and presumably act as regulators of growth and development in determining auxologic choices between homeostasis and plasticity [8]–[10].

Already applied in biomedical areas such as genetics, molecular biology, microbiology, and epidemiology, networks often have revealed surprising and unanticipated biological and functional insights, delineating the possibility of a new, holistic approach in scientific investigation. This approach ideally aims to define and analyze the interrelationship of *all* the elements in a biomedical system in order to understand how a system works in ever changing conditions (a new discipline called “*Systems Biology*”) [11]. An apparently more modest but not less important task is to apply such an approach to the ordinary data sets used in medical practice; in particular, we will produce an example based on standard orthodontic data.

Network analysis has been applied recently to orthodontics to detect and visualize the most interconnected clinical, radiographic, and functional data pertaining to the orofacial system [12]. In particular, by considering phenotypic, functional, and radiographic characteristics it has been shown that different kinds of dentofacial malocclusions correspond to different network structures (a malocclusion is a misalignment of teeth or incorrect relation between the teeth of the two dental arches).

**Figure 1:**
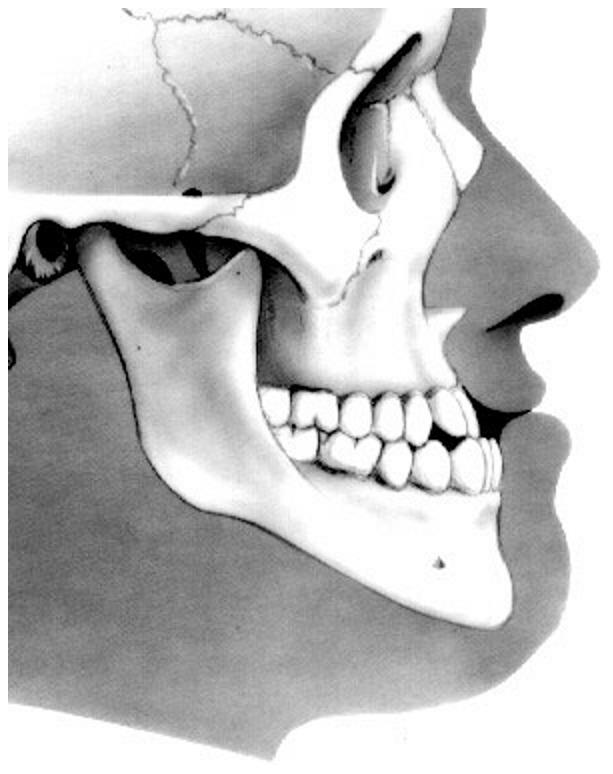
Class III malocclusion with protrusion of the lower dental arch.

During the diagnostic process to establish the objectives, strategies, priorities and sequences of treatment, the orthodontist has to identify and locate the critical points of malocclusion [13]. Among malocclusions, the more severe is the so-called Class III malocclusion, often associated with the protrusion of the lower dental arch (Fig. 1). Class III malocclusion in growing subjects is characterized by a complex combinations of skeletal features (*e.g.* a shorter and more retrusive maxilla, an excess of lower anterior face height, a shorter anterior cranial base length, a more acute cranial base angle) with multiple dentoalveolar compensatory processes (*e.g.* proclined maxillary incisors, retroclined mandibular incisors) [14]–[18]. The management of the architectural and structural Class III network parameters forces the orthodontist to collect clinical and radiographic data sets on craniofacial characteristics, growth, and function. The paradox of daily orthodontic practice is that these data sets may bring more disorientation than understanding of the main problem of the patient [19]. With the aim of identifying pathognomonic traits of severity for Class III malocclusion, Freer [20] found that labiolingual spread and overjet were the most critical variables, while Stellzig-Eisenhauer et al [19] focused attention on the individualized combination of palatal plane angle, inclination of lower incisors, and Wits appraisal, but no morphologic trait was shown to be indicative of potential Class III development [20]–[23].

The craniofacial region can be regarded as a complex system that grows and remodels itself following an intricate network of auxologic forces, distortive processes and/or compensatory mechanisms [18]. Complex systems are dynamic systems that present with the capacity of self-organizing a large number of interacting elements in a non-linear fashion (*e.g.* forests, ants, flocks of birds, financial markets, the immune system) [3]. In order to understand the function of a biological organization it often is beneficial to conceptualize it as a systems of interacting elements and to define the dynamic behavior of these components [1], [11]. The global behavior of complex systems cannot be explained solely on the basis of a single physical law, or the behavior of individual elements. The cooperation of the elements determines the overall behavior and provides properties that can be totally unrelated to the individual components of the system (“more is different”). The system must be analyzed in its entirety, as a coherent unit: it is pattern that matters [24], [25].

The aim of this study is to show how “network thinking” and network modeling leads to a systemic analysis of standard diagnostic data under a different perspective that digs out previously undiscovered information. In particular, we will identify the physiological and/or pathological characteristics in a large cross-sectional sample of 532 female Class III subjects on the basis of a model derived from network analysis.

## Methods

### Objectives

The aim of this study is to apply conjunctly statistical analysis with network tools and methodologies to Class III malocclusion features' longitudinal (i.e. time varying) datasets in order to uncover the systemic importance of such features and to individuate the possible emergence of features' subset driving the orofacial development of Class III malocclusion

### Participants

This study analyzed the pretreatment lateral cephalometric records of 532 untreated Class III Caucasian female patients collected from the Department of Orthodontics of the University of Florence, Italy, and from the Graduate Orthodontic Program at the University of Michigan, Ann Arbor, Michigan. All these subjects was enrolled previously in large descriptive estimates of craniofacial growth in Class III malocclusion [14], [23], [26]. The age range was between 6 years 4 months to 17 years 3 months.

To be included in this study, the female patients had to satisfy all of the following inclusion criteria:

Caucasian ancestry;
no orthopedic/orthodontic treatment prior to cephalogram;
diagnosis of Class III malocclusion based on anterior cross-bite, accentuated mesial step relationships of the primary second molars, permanent first molar relationship of at least one half cusp Class III;
no congenitally missing or extracted teeth.

### Description of Procedures or Investigations undertaken

The subjects were examined separately in four age groups: Group G1 (from 7 to 10 years) 240 subjects, Group G2 (from11 to 12 years) 89 subjects, Group G3 (from 13 to 14 years) 105 subjects, and Group G4 (from 15 to 17 years) 98 subjects.

**Figure 2:**
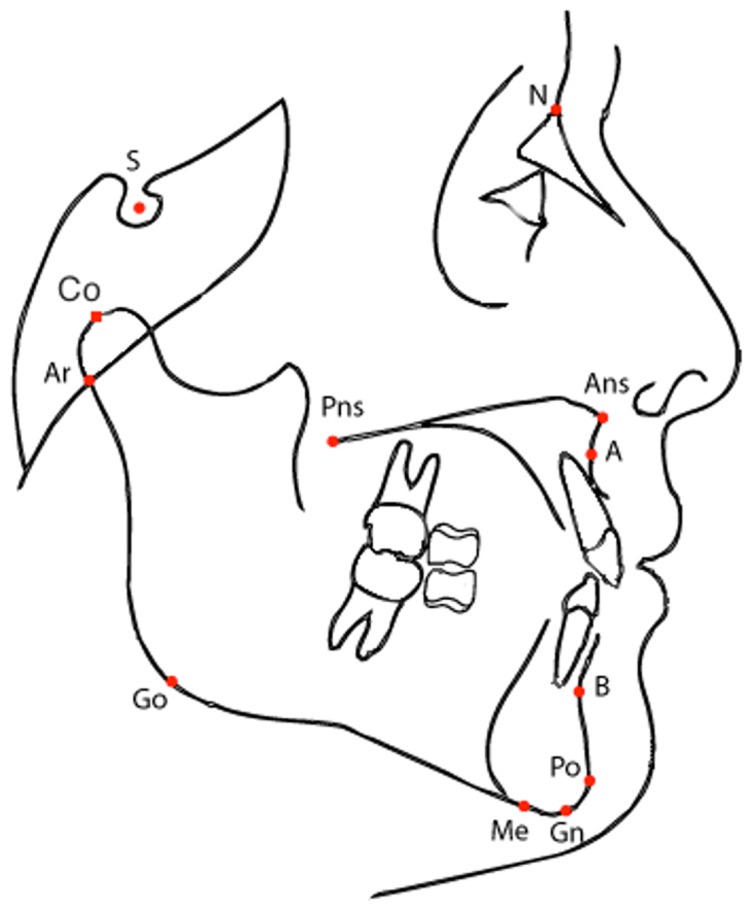
Cephalogram reference points. Most of the cephalometric landmarks are either angles or normalized linear distances. As an example, SN-GoGn is an angle between anterior cranial base and mandibular plane. The 21 cephalometric landmarks analyzed in the paper correspond to the standard set of features analyzed in orthodontics (see Table 1).

The cephalometric analysis required the digitization of 21 landmarks on the tracing of each cephalogram (Fig. 2). The error of the method for the cephalometric measurements was evaluated by repeating the measures in 100 randomly selected cephalograms. Error was on average 0.6° for angular measures and 0.9 mm for linear measures.

### Ethics

All data used in this study have been previously published as referenced in the methods section. Written informed consent was obtained from the patients' parents as part of their orthodontic treatment. The approval of an Ethical Committee was not sought as all data analysed were collected as part of routine orthodontic pre-treatment diagnosis.

### Statistical methods

We analize the correlation matrices among the 21 cephalographic landmarks considered by using complex netwoks. First we calculate (using KNIME [27]) for each pair of features their sample Pearson correlation coefficient r_xy_ = (<xy>-<x><y>)/s_x_ s_y_, where x,y are the numerical values of the landmarks, <…> indicates sample means and s_x_, s_y_ are their sample standard deviations. Each r_xy_ can be considered as the weight of a link between x and y; the associated network therefore is a complete graph, *i.e.* a network where every node is connected to every other node. Such correlation graphs already have been considered in other applications of network theory, like finance and genomics [28], [29]; in order to dig out the information present in the whole correlation matrix and sort out relevant features with their global correlations, some filtering has to be applied. Our choice is to use a cutoff to correlation values in order to consider only the most significant correlations [12]; therefore we consider two features (the nodes of our graphs) to be linked if |r_xy_|>0.40. Notice that at difference with most previous studies in networks, we do not discard negative correlations: this is a critical point when analyzing any complex systems where important relations, as a negative feedback, naturally would show up as significantly high negative correlations.

Networks have been visualized using the software yEd [30] with the standard layout; the choice of filtering at |r_xy_|>0.40 reduces the complexity of the system and permitted the identification of many characteristics just by visual inspection. In particular, it is very easy to identify *bridge nodes*, i.e. nodes whose absence would split the graphs in two or more separate parts. Bridge nodes are important both because they allow to detect separate subsystems (sets of highly correlated features) and because they represent the connection among such subsystems.

Furthermore, to investigate the presence of functional modules, we have searched for motifs in our filtered networks. Motifs searches are potentially valuable tools to predict unknown interactions involving 3–5 nodes (rarely more than 6). These organized sets of interactions are capable of higher order functions (such as amplification), and hence probably represent the functional capabilities within the network. They provide balance between modules through signaling gates (*i.e.* negative feed-forward motifs), favoring plasticity (open-gate configuration), or homeostasis (closed- gate configuration). We have focused on the presence of *clicques* (subsets where each of node is connected to every other node) as they naturally represent the presence of a subsystem acting as a whole: in fact, every feature in such a subsystem is interrelated. To individuate the presence of clicques, we have employed the Power Graphs plugin [31] in the software Cytoscape [32].

## Results

**Figure 3:**
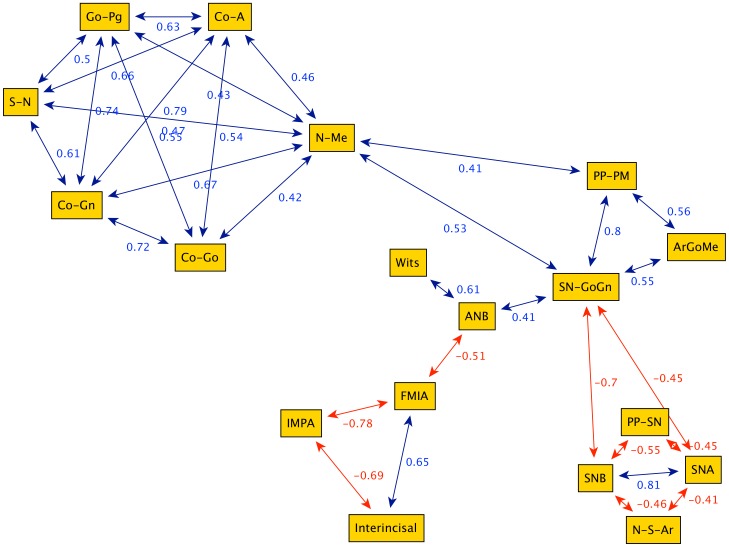
Graph obtained from the cephalometric data of 240 female Class III patients between 7 and 10 years of age (group G1). The highly connected nodes N-Me (anterior facial height) and SN-GoGn (divergence between the anterior cranial base and mandibular body) work as bridges, i.e. they connect separate sub-graphs. The graph highlights a division between the cephalometric parameters: linear (upper left nodes: Go-Pg, Co-A, S-N, Co-Gn, Co-Gn, N-Me), angular parameters (upper right: PP-PM, SN-Go-Gn, Ar-Go-Me) and adaptive dentoalveolar parameters (lower left: IMPA; FMIA, Interincisal).

**Figure 4:**
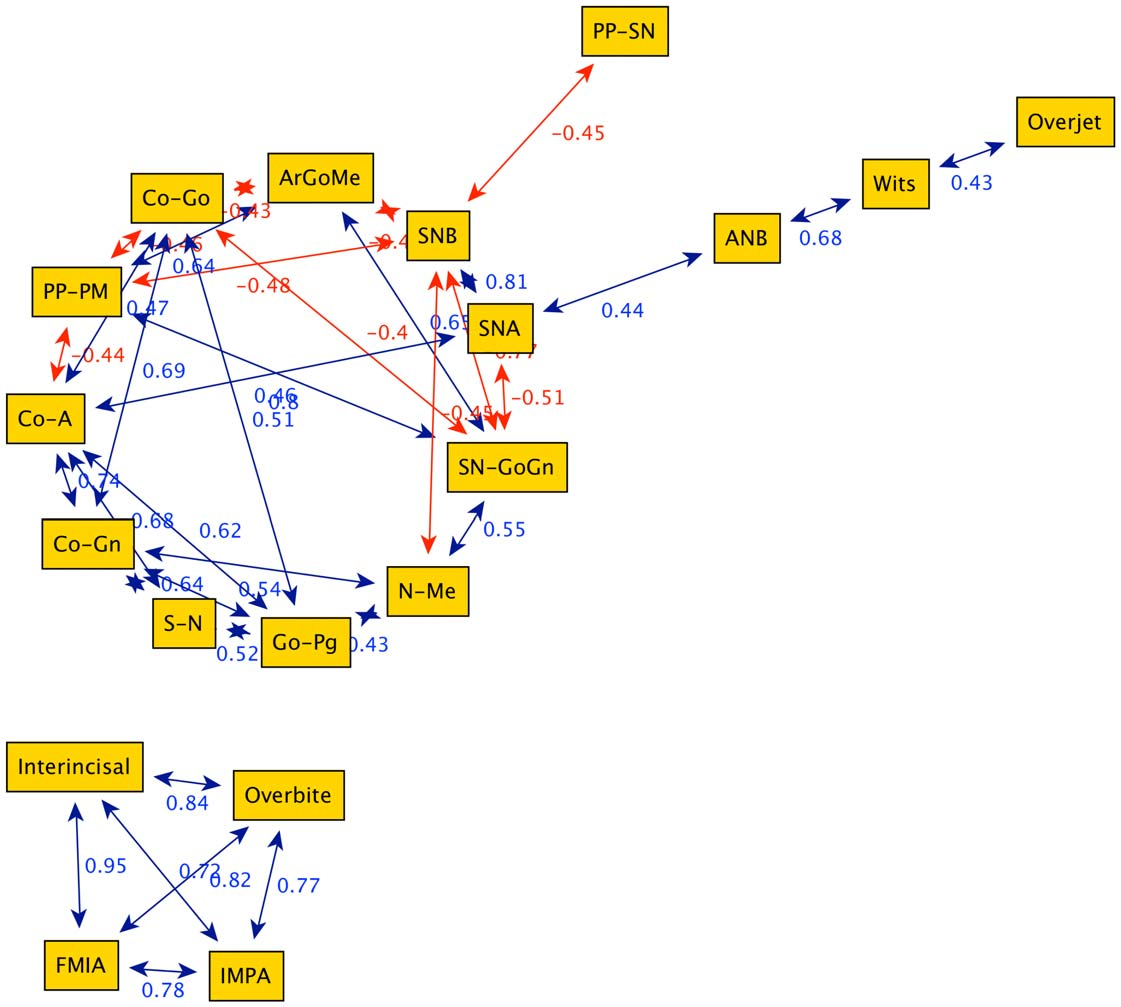
Graph obtained from cephalometric data of 90 female Class III patients between 11 years and 12 years of age (group G2). The graph is composed by two characterized groups: structural (upper group) and dentoalveolar adaptive (lower group of four nodes).

**Figure 5:**
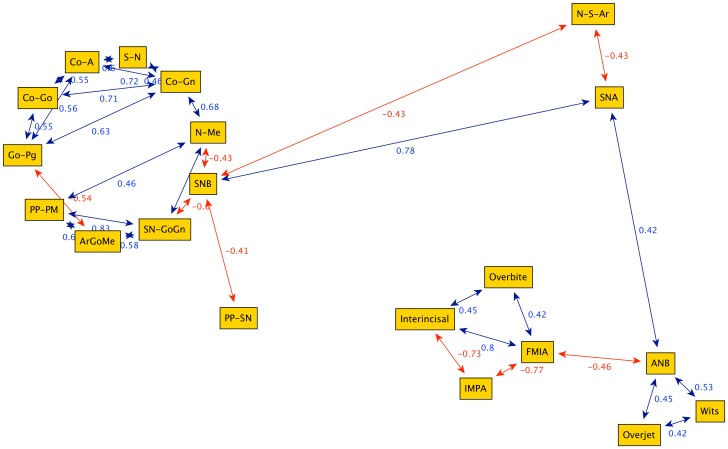
Graph obtained from cephalometric data of 105 female Class III patients between 13 and 14 years of age (group G3). The main bridge node is S-N-B (longitudinal position of the maxillary arch) divides the structural nodes from the ones representing dentoalveolar adaptive and mixed features.

**Figure 6:**
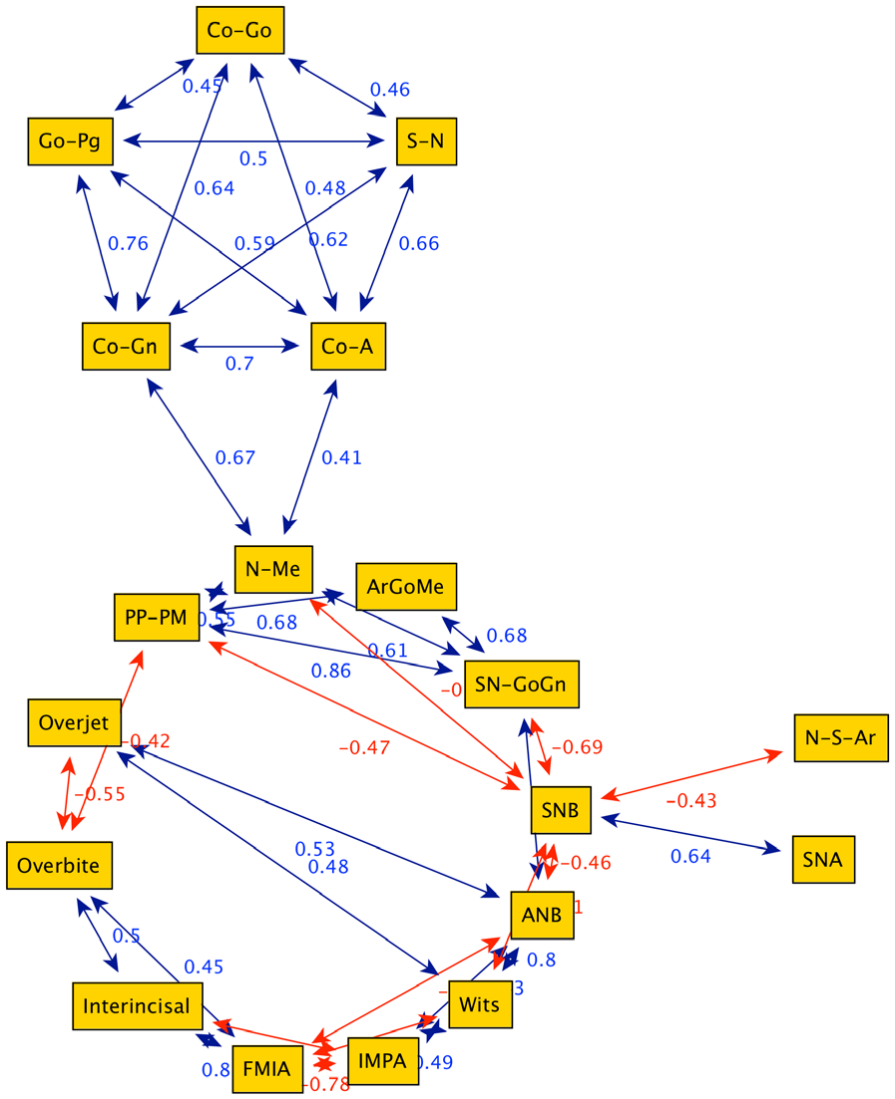
Graph obtained from cephalometric data of 99 female Class III patients between 15 and 17 years of age (group G4). The graph is divided into two groups clearly inter-connected via the bridge N-Me (anterior facial height).

Figures 3, 4, 5, 6 illustrate the correlation networks of the cephalometric characteristics of 532 Class III female patients, from 7 to 17 years of age. In all ages considered, the most-connected nodes are those related to vertical skeletal features (N-Me, SN-GoGn, PP-PM). These vertical parameters always are connected with those of mandibular sagittal nodes (SNB, GoPg). These strong patterns of interaction are observed for all ages considered. Due to the persistence of such network topology in all age groups, these highly connected nodes can be regarded as the key features in the growth of female Class III subjects.

**Figure 7:**
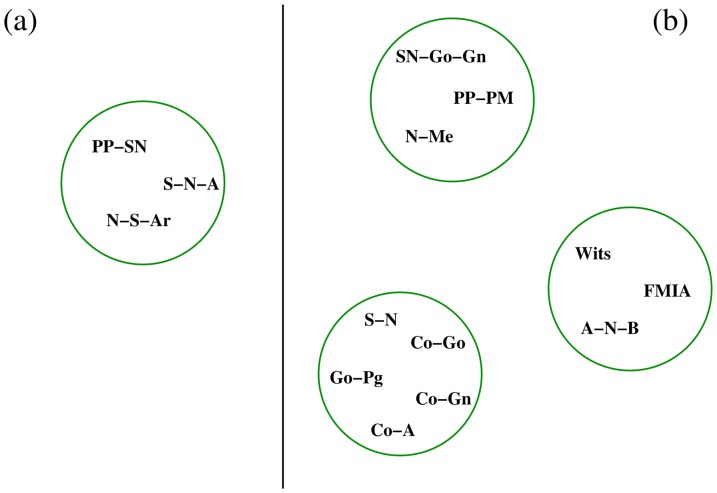
Cliques (motifs) individuated by the Power Graphs analysis for female patients (15–17 years) with mild Class III malocclusion (panel A) and with severe Class III malocclusion (panel B). Mild Class III patients show a single clique of only three structural nodes (SNA, N-S-Ar, PP-SN). Severe Class III patients show the presence of three separate cliques: mandibular sagittal nodes (S-N,…), maxillomandibular divergence nodes (N-Me,…) and adaptive nodes (Wits,..). The comparison between the two figures indicates that severe Class III patients are characterized by the presence of groups of strongly inter-correlated features, i.e. tend to act as a single whole system.

Further results come from the Power Graph analysis of the networks. With the aim of defining the possible clinical relevance of these orthodontic network patterns, the patients were differentiated into two cephalometric categories using Wits appraisal of jaw disharmony, a simple method whereby the severity of degree of anteroposterior jaw displasia may be measured on a lateral cephalometric head film. The two class consist of “mild” and “severe” Class III patients (Wits appraisal greater than −3 mm and Wits appraisal smaller or equal to −3 mm, respectively) [19]. The visual network inspection of these “mild” and “severe” Class III patients (group G4) reveals several interesting characteristics:

1. the networks of the “mild” patients exhibits a balanced node pattern (Fig. 7a);
2. in the “severe” patients group, we find a preponderance of maxillomandibular divergence nodes (related to the vertical development of the craniofacial system) and mandibular sagittal nodes (related to the horizontal prominence of the chin), with poor balance of adaptive nodes (Fig. 7b).

## Discussion

Instead of searching single or multiple dentoskeletal radiographic predictors variables, our work attempts to delineate the overall dentofacial organizing principles, the functional dynamics, and the regulatory growth principles of Class III malocclusion. The cephalometric data of a large retrospective cohort of 532 Class III female subjects, in mixed and permanent dentition, were analyzed through a combination of multivariate computational techniques: networks analysis of correlation matrices and search for regulatory motifs. These high-throughput techniques allow the extraction and identification of new biological insight from data regarding several related topics of importance during the Class III craniofacial growth such as robustness, adaptation, time progression, and structural stability.

Understanding structure-dynamics relationships in networks is a major goal of complex system research. In several biomedical fields, the analysis of interaction dynamics of the components may be useful to capture the essential behavior of the system, to understand higher-order biological function, and also to facilitate prediction responses [9], [25].

General principles behind the relationships between orthodontic structure and dynamics still are lacking, in part due to the scarcity of sufficiently general formalism to study structure and dynamics within a common framework. When a complex system is investigated using network analysis, the network map often shows groups of nodes only weakly connected, alternating with groups of highly connected nodes. Many aspects of the inherent complexity of nature follow a pattern that is the same in many contexts (from biology to ecology, sociology, financial markets, etc.). Among the network of connections, very few nodes have many links (“*hubs*”), while the majority of the remainder are characterized by few or very few links. These hubs govern the entire system through preferential interactions, facilitating the movement of information, creating shortcuts between distant nodes, helping to create a robust network (“*small world networks*”) that can adapt to environmental stresses [4], [24].

A previous investigation illustrated the more compact network Class III malocclusion structure as regard to Class I and II when considering phenotypic, functional, and radiographic characteristics [12]. The results of the current study showed that, in the interrelationship of Class III skeletal elements, the “*driver nodes*” that presumably guide the growth of the orofacial system are located in the interplay between maxillomandibular divergence (PP-PM, NS-Go-Gn) and mandibular sagittal nodes (Go-Gn, Co-Gn). This structural organization, reflected in the network topology, probably constrains the range of dynamical behaviors available to the system during the generative process of the malocclusion. Our data confirm the observation of Bui et al. [21] regarding the generative process of Class III malocclusion observed in a retrospective cohort of 309 patients: the most important cephalometric variables reflect the anteroposterior and vertical imbalance during growth, rather than specific Class III craniofacial structures.

Malocclusions are isoforms of biological complexity. The network of functional and morphologic characteristics of the orofacial system causes diffuse connections of strict interdependence. Any therapeutic intervention applied to a part of the system, invariably has an impact on other structures. For example, the decision to open the bite by rotating the mandible clockwise must take into account the concomitant effects on the vertical dimension, on the convexity of face, and on the potential divergence of the occlusal plane [13], [17].

Once the pattern of a malocclusion has been identified, it becomes easier to analyze the force flow in the orofacial network, to define the local functional entities involved (in Graph theory, *motifs*) and localize signaling gates that provide among between modules, rather than taking solely into consideration the morphological characteristics of the system [8].

The present study shows that during the growth process of Class III malocclusion the skeletal vertical and sagittal growth features (SN-GoGn, PP-PM) are central in the interacting network of the system components: these nodes can be considered the “driver nodes” for the growth of the orofacial system. The ability of the orofacial system to function as an integrate unit may arise from the balance of activities between the modules: this may be the core design principle revealed by orthodontic network analysis. Network analysis revealed that the patients with more unbalanced cephalometric features (“severe patients”) present a network topology with a preponderance of the skeletal nodes and minor relevance of adaptive dentoalveolar nodes. In the “mild” patient group, the network topology showed a greater balance between skeletal and adaptive craniofacial features. In the patients with more pronounced radiographic Class III features, we have identified two subnetworks of strong functional interaction (cliques). As observed in several metabolic pathways, these subnetworks are recognized as critical elements of biological organization [7], [8]; they work as feed-forward loops, with high capacity of anticipatory regulation as opposed to the homeostatis effects of feed-back loops. Such analysis confirms the importance of considering the co-occurrence of the interrelated morphologic features, reinforcing the hypothesis that these sites of co-occurrence of the overall interrelated morphologic features are more suitable to indicate the favorable or unfavorable progression of this type of disharmony respect to the individual orthodontic features. Presumably, the convergence of the orthodontic therapeutic approaches into these modules allows the clinician to maximize results and to shorten treatment times.

Computational technology has proved to be most useful in the handling of mass data (in the present case, a set of cephalometric measurements). As orthodontic studies shift from local description to system analysis, we need to identify the design principles of large craniofacial features networks. The limitations of viewing the head region in two dimensions only are well known. However, postnatal growth differences and the high incidence and magnitude of anteroposterior and vertical dentofacial abnormalities render this record useful for characterizing the overall morphology of the growing orofacial system.

The result of the present study indicate that, in their most basic abstraction level, the orofacial radiographic characteristics can be represented as networks using nodes to represent orthodontic characteristics, and edges to represent their various types of interactions. A substantial portion of the Class III issues during growth is driven by only a few nodes. By linking radiographic data and phenotypes to clinical characteristics in a causal or correlative manner, these observations may contribute to the construction of a model that provides a theoretical framework of the reciprocal interaction between organizing craniofacial pathways, growth, and malocclusion.

In conclusion, due to their generality, the application of network mathematical models could increase the interpretation of quantitative, patient-specific information and help to better targeting of therapy not only in orthodontics but also in other medical fields.

**Table 1:** The 21 cephalometric variables employed in our study.

SN	anteroposterior length of the cranial base
Wits	Wits appraisal
Co-A	midfacial length as distance from Co to A
Co-Gn	mandibular length as distance from Co to Gn
Ar-Go	mandibular ramus height
NS-GoGn	divergence of the mandibular plane relative to the anterior cranial base
NS-Ar	saddle angle
ArGoMe	gonial angle
SNA	anteroposterior maxillary position to the anterior cranial base
SNB	anterorposterior mandibular position to the anterior cranial base
IMPA	angle between the lower incisor with the mandibular plane
ANB	anteroposterior relation of the maxilla and the mandible
Interincisal	angle between the axis of the upper and the lower incisor
PP-SN	inclination of the palatal plane in relation to anterior cranial base
PP-PM	inclination of the palatal plane in relation to the mandible plane
NMe	anterior facial height
FMIA	angle between the axis of the lower incisor and the Frankfort plane
Overbite	Vertical distance between the incisal edges of the most protrusive maxillary and mandibular central incisors.
Overjet	Horizontal distance between the incisal edge of the most protrusive maxillary central incisors and the most facial aspect of the crown of the most protrusive mandibular central incisor
Go-Pg	distance between gonion and pogonion points
Co-Go	distance between condylion and gonion points

Most of the cephalometric variables are angles or distances derived from the cephalometric reference landmarks (Fig. 2).
